# Clinical safety of ProMRI implantable cardioverter-defibrillator systems during head and lower lumbar magnetic resonance imaging at 1.5 Tesla

**DOI:** 10.1038/s41598-019-54342-4

**Published:** 2019-12-03

**Authors:** Wolfgang Rudolf Bauer, Dennis H. Lau, Christian Wollmann, Andrew McGavigan, Jacques Mansourati, Theresa Reiter, Simone Frömer, Mark E. Ladd, Harald H. Quick

**Affiliations:** 1 Department of Internal Medicine I, Universitätsklinikum Würzburg, Würzburg, Germany; 2 Centre for Heart Rhythm Disorders, South Australian Health and Medical Research Institute, University of Adelaide and Royal Adelaide Hospital, Adelaide, Australia; 3 Department of Internal Medicine III, Universitätsklinikum St. Pölten, St. Pölten, Austria; 4 Institute of Cardiovascular Research, Karl-Landsteiner Society, St. Pölten, Austria; 5 Flinders Medical Centre, Bedford Park, Australia; 6 Hôpital de la Cavale Blanche, University Hospital of Brest and University of Western Brittany, Brest, France; 7 Centre for Clinical Research, BIOTRONIK SE & Co. KG, Berlin, Germany; 8 Medical Physics in Radiology, German Cancer Research Center, Heidelberg, Germany; 9 Faculty of Physics and Astronomy and Faculty of Medicine, University of Heidelberg, Heidelberg, Germany; 10 High-Field and Hybrid MR Imaging, University Hospital Essen, Essen, Germany; 11 Erwin L. Hahn Institute for MR Imaging, University Duisburg-Essen, Essen, Germany

**Keywords:** Cardiology, Engineering

## Abstract

Magnetic resonance imaging (MRI) has long been contraindicated in patients with implanted pacemakers, defibrillators, and cardiac resynchronisation therapy (CRT) devices due to the risk of adverse effects through electromagnetic interference. Since many recipients of these devices will have a lifetime indication for an MRI scan, the implantable systems should be developed as ‘MRI-conditional’ (be safe for the MRI environment under predefined conditions). We evaluated the clinical safety of several Biotronik ProMRI (‘MRI-conditional’) defibrillator and CRT systems during head and lower lumbar MRI scans at 1.5 Tesla. The study enrolled 194 patients at 22 sites in Australia, Canada, and Europe. At ≥9 weeks after device implantation, predefined, non-diagnostic, specific absorption rate (SAR)-intensive head and lower lumbar MRI scans (total ≈30 minutes per patient) were performed in 146 patients that fulfilled pre-procedure criteria. Three primary endpoints were evaluated: freedom from serious adverse device effects (SADEs) related to MRI and defibrillator/CRT (leading to death, hospitalisation, life-threatening condition, or potentially requiring implanted system revision or replacement), pacing threshold increase, and sensing amplitude decrease, all at the 1-month post-MRI clinical visit. No MRI-related SADE occurred. Lead values remained stable, measured in clinic and monitored daily by the manufacturer home monitoring technology.

## Introduction

Magnetic resonance imaging (MRI) has long been contraindicated in patients with pacemakers and implantable cardioverter-defibrillators (ICDs) due to the risk of adverse effects through electromagnetic interference^1,2^. Since 50% to 75% of pacemaker or ICD patients will likely require MRI during their lifetimes due to the high probability of comorbidities such as stroke, lumbar disease, arthritis, or cancer, efforts have been made to understand the underlying mechanisms responsible for adverse events, to develop guidelines, and to introduce technical advances allowing these patients to undergo MRI safely^1^. The industry was called upon to design all components of cardiovascular implantable electronic systems to be suitable for current and evolving MRI technologies^1,2^.

Accordingly, several pacemaker and ICD models have been developed as MRI-conditional and were demonstrated safe for the MRI environment under predefined conditions^1,3–13^. However, recent literature^14^ indicates that MRI scans are often being denied even to patients with MRI-conditional devices. Reasons reported by departments not offering the scans included residual concerns regarding risk (≈50%) and lack of an evidence base for safety (≈15%) (cf. Fig. 3 in^14^). Another study^15^ indicates that many scans are denied in patients with a clinical indication and that the presence of an MRI-conditional system does not seem to increase the rate of MRI procedures. In light of these reports, it appears critical to increase the scientific literature on the safety and rate of adverse effects during MRI scans of patients with MRI-conditional cardiac devices.

Complex physical interactions between the electronic implants and the MRI environment necessitate testing on new pulse generator and lead combinations^1,9^, especially of MRI-conditional ICD or cardiac resynchronisation therapy (CRT) defibrillator (CRT-D) and CRT pacemaker (CRT-P) systems^1,9^, for which the available data are limited to a few device models^8,10–12^. The present study was designed to provide supporting evidence for the clinical safety of several MRI-conditional ICD and CRT-D/-P systems (Biotronik SE & Co. KG, Berlin, Germany) during non-diagnostic head and lower lumbar MRI scans at 1.5 Tesla (T).

## Methods

The ProMRI PROVEN master study (Master Study for the MRI Compatibility of the Solia S Pacing Lead, the Linox^smart^ ProMRI ICD Lead, and the Corox ProMRI OTW Coronary Sinus Lead in Combination with the Ilesto/Iforia ICD or the Evia/Entovis Triple Chamber Pacemaker) was a multicentre, prospective, single-arm, non-randomised investigation. The study was performed in compliance with good clinical practice guidelines and the Declaration of Helsinki. The study protocol was approved by the ethics committee of the University of Würzburg, Germany (Institute of Pharmacology and Toxicology). All patients gave written informed consent before enrolment (ClinicalTrials.gov Identifier: NCT01809665. Study registered on March 13, 2013).

### Study participants

Patients were enrolled if they had a standard indication for an ICD, CRT-D, or CRT-P and were able and willing to complete all testing required by the clinical protocol including one non-clinically indicated MRI scan. Patients were not enrolled in case of presence of metallic objects in the patient’s body susceptible to interaction with MR, or cardiovascular implants that contradict the conditions of the current Biotronik ProMRI® manual. Detailed inclusion and exclusion criteria are provided in the Supplementary Information File.

### ICD, CRT-D, and CRT-P systems studied

After enrolment, patients underwent implantation of a single-chamber or dual-chamber ICD, an ICD DX (atrial sensing via ICD lead)^16^, a CRT-D, or a CRT-P according to the individual indication. Investigators could choose between the pulse generators and leads listed in Table 1, in any of the combinations allowed in the technical manuals. All combinations used were CE-approved as MRI-conditional (labelled ProMRI®) before their inclusion in the study. The CE-mark for MRI conditionality of these products was based on *in-vitro* and technical validation tests and literature data and not on clinical data. A post-market observational study such as ours was necessary from a regulatory perspective to provide supporting evidence for the clinical safety.

**Table 1:** Investigated ProMRI devices.

Device type	Connector^a^	Device models
**Pulse generator**		
Single-chamber ICD	DF-1 or DF-4	Ilesto 5, Ilesto 7, Iforia 5, and Iforia 7 (VR-T)^b^
Dual-chamber ICD	DF-1 or DF-4	Ilesto 5, Ilesto 7, Iforia 5, and Iforia 7 (DR-T)^b^
ICD DX	DF-1	Ilesto 5, Ilesto 7, Iforia 5, and Iforia 7 (VR-T DX)^b^
CRT-D	DF-1 or DF-4	Ilesto 5, Ilesto 7, Iforia 5, and Iforia 7 (HF-T)^b^
CRT-P	—	Evia and Entovis (HF-T)^b^
**Lead category**^**c**^		
ICD lead with DF-1	DF-1	Linox^smart^ ProMRI S (65, 75)^d^
		Linox^smart^ ProMRI SD (65/16, 65/18, 75/18)^e^
		^f^Linox^smart^ ProMRI S DX (65/15, 65/17)^e^
ICD lead with DF-4	DF-4	^g^Protego ProMRI SD (65/16, 65/18, 75/18)^e^
		^h^Protego ProMRI S (65, 75)^d^
Solia	—	Solia S (45, 53, 60)^d^ (atrial and ventricular leads)
Corox	—	Corox ProMRI OTW (75 BP, 85 BP)^d^
		Corox ProMRI OTW-L (75 BP, 85 BP)^d^
		Corox ProMRI OTW-S (75 BP, 85 BP)^d^

^a^DF-4 connector (four-pole inline) is a new standard since 2010, gradually replacing the bulkier DF-1 connector (bi- or trifurcated) in ICDs/CRT-Ds^17^.

^b^Suffix “-T” in pulse generator name indicates Biotronik Home Monitoring® function.

^c^For the primary endpoint 1, leads were grouped into these four categories. ICD and Solia leads were screw-in. Corox coronary sinus leads for CRT-D/-P devices (in addition to atrial lead and ICD or ventricular lead) have a pre-shaped distal section and are positioned by an over-the-wire or stylet-driven technique.

^d^Length of the lead in cm.

^e^Length of the lead in cm/ distance of proximal shock coil to lead tip in cm.

^f^Lead for the ICD DX.

^g^Previously labeled as Linox^smart^ ProMRI DF4 SD (65/16, 65/18, 75/18).

^h^Lead added to the study by later protocol amendments and available for European sites only.

BP: bipolar; CRT-D/-P: cardiac resynchronisation therapy defibrillator/pacemaker; DX: ICD lead allowing atrial sensing via floating dipole^16^; ICD: implantable cardioverter-defibrillator; MRI: magnetic resonance imaging; ProMRI: MRI-conditional.

The ProMRI pulse generators were designed with minimised ferromagnetic and paramagnetic material to reduce the mechanical torque and force in strong magnetic fields, and with electronic circuits and software components capable of better rejecting electromagnetic interference than non-MRI-conditional devices. The ProMRI leads are designed to limit current induction. An MRI mode was implemented to provide pacing without the risk of inappropriate inhibition by the scanner’s electromagnetic fields and to eliminate functional interference by deactivation of anti-tachycardia therapy and other features during the MRI procedure^7–9^. All devices offered the Home Monitoring option.

The investigated ProMRI leads were endocardial and bipolar, with steroid-eluting, fractally-coated iridium electrodes. The ICD leads were from the Linox^smart^ ProMRI (DF-1 connector) and Protego ProMRI (DF-4 connector)^17^ families and had one shock coil (S models), two shock coils (SD models), or one shock coil and a floating atrial sensing dipole (S DX). The right atrial (RA) and right ventricular (RV) pacing leads were from the Solia S family, and coronary sinus leads from the Corox ProMRI OTW family (Table 1).

### Study procedures

**Figure 1:**
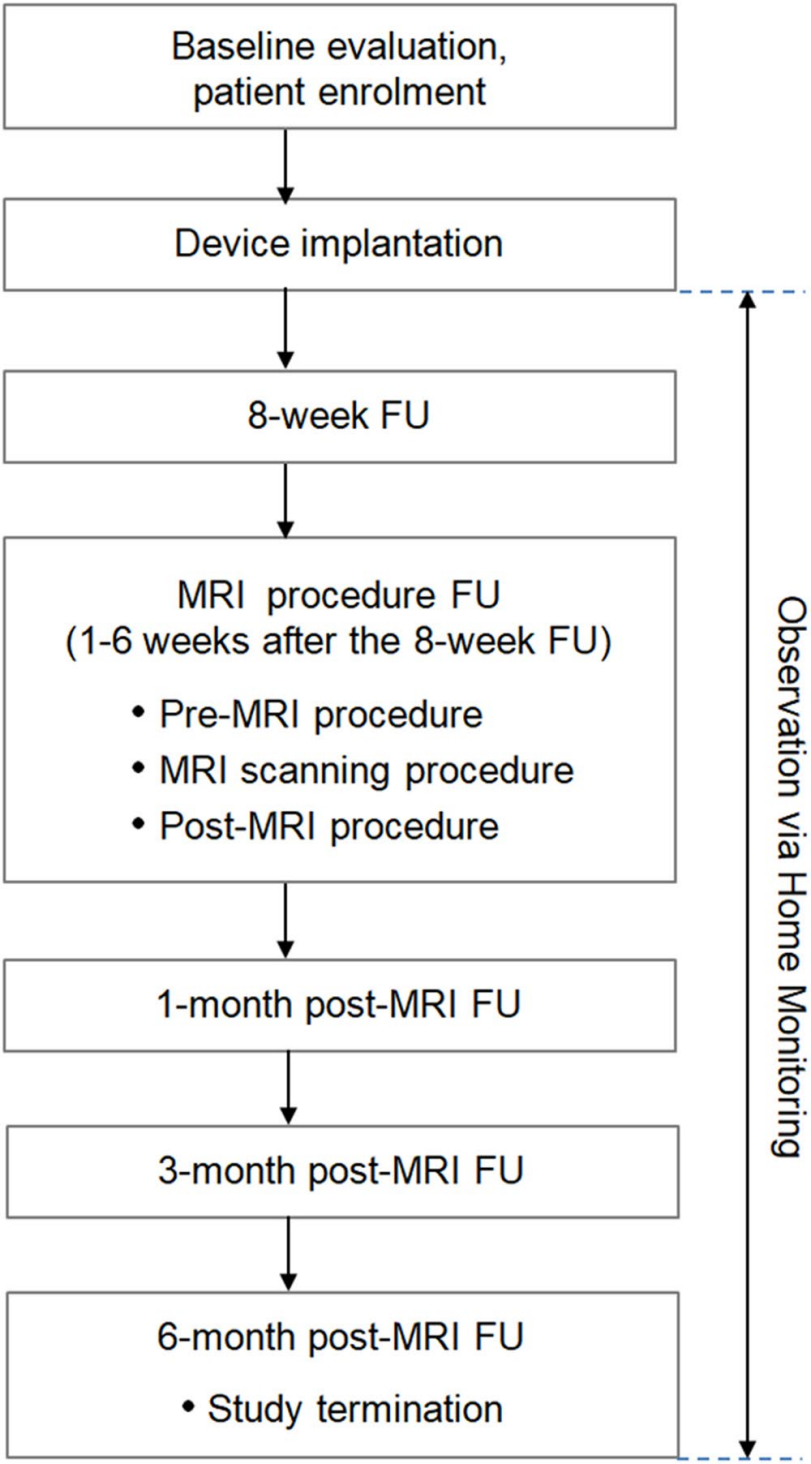
Flow chart of the study. FU: follow-up; MRI: magnetic resonance imaging.

Study timelines are summarised in Fig. 1. All devices were interrogated in-house after implantation, at the 8-week follow-up, at the MRI procedure follow-up (immediately before and after MRI), and at the 1-, 3-, and 6-month post-MRI visits. All adverse events were recorded. The Home Monitoring option was used to collect pacing thresholds and sensing measurements and to monitor arrhythmia episodes automatically on a daily basis.

Immediately before study-specific MRI scans, the pulse generators were programmed into one of the available MRI modes to disable detection and therapy of ventricular tachyarrhythmia. Pacing was programmed to an asynchronous mode (V00/D00) or “OFF”. The MRI mode was chosen at the investigator’s discretion. During MRI scans, patients were continuously monitored by pulse oximetry, ECG, and/or blood pressure unit.

The MRI scans consisted of head and lower lumbar MRI, two of the most common MRI examinations in medical practice. Siemens, Philips, and GE MRI systems were used, with cylindrical magnets and a static magnetic field strength of 1.5 T. Scanning sequences were predefined to ensure comparability between imaging sequences at different sites and MRI systems (Table 2), and to evaluate the implanted systems at the maximum allowed burden according to labelling conditions (footnote “a” of Table 2). Total MRI scan time per patient was close to but did not exceed 30 minutes, comprising 8–9 sequences for the head (≈16 minutes) and 6–7 sequences for the lumbar scan (≈14 minutes).

**Table 2:** Predefined scan sequence types^a^.

Body region	Scan sequence types		
	Siemens	Philips	GE
Head	3-plane localiser	3-plane localiser	3-plane localiser
(landmark on eyes)	SAG SE T1	Reference scan	ASSET calibration
	AX TSE T2	SAG SE T1	SAG SE T1
	T2 TIRM	AX FSE T2	AX FSE T2
	Diffusion	T2 FLAIR	AX T2 FLAIR
	3D TOF MT	Diffusion	AX diffusion
	CE-MRA	3D TOF MT	AX 3D TOF MT
	Perfusion	CE-MRA	CE-MRA
		COR FSE T2	AX perfusion
Lumbar	Localiser	Localiser	Localiser
(landmark on trochanter)	SAG T1	Reference scan	SAG T1
	SAG T2	SAG T2	SAG T2
	AX T1	COR T2	AX T1
	AX T2	SAG T1	AX T2
	SAG diffusion	AX T2	SAG T1
		STIR	

^a^The maximum slew rate of the MRI scanners’ gradient fields did not exceed 200 T/m/s per axis according to the study protocol. Specific absorption rate (SAR) could not exceed 2 W/kg for the body and 3.2 W/kg for the head. All MRI scans were performed in the “normal scanning mode”. MRI angiography and perfusion sequences were performed without contrast.

3D: three-dimensional; ASSET: array spatial sensitivity encoding technique; AX: axial; CE: contrast enhanced; COR: coronal; FLAIR: fluid attenuated inversion recovery; FSE: fast spin echo; GE: General Electric; MRA: magnetic resonance angiography; MRI: magnetic resonance imaging; MT: magnetisation transfer; SAG: sagittal; SE: spin echo; STIR: short tau (inversion time) inversion recovery; T1: T1-weighted (short repetition time and short echo time sequence); T2: T2-weighted (long repetition time and long echo time sequence); TIRM: turbo inversion recovery magnitude; TOF: time of flight; TSE: turbo spin echo.

**Figure 2:**
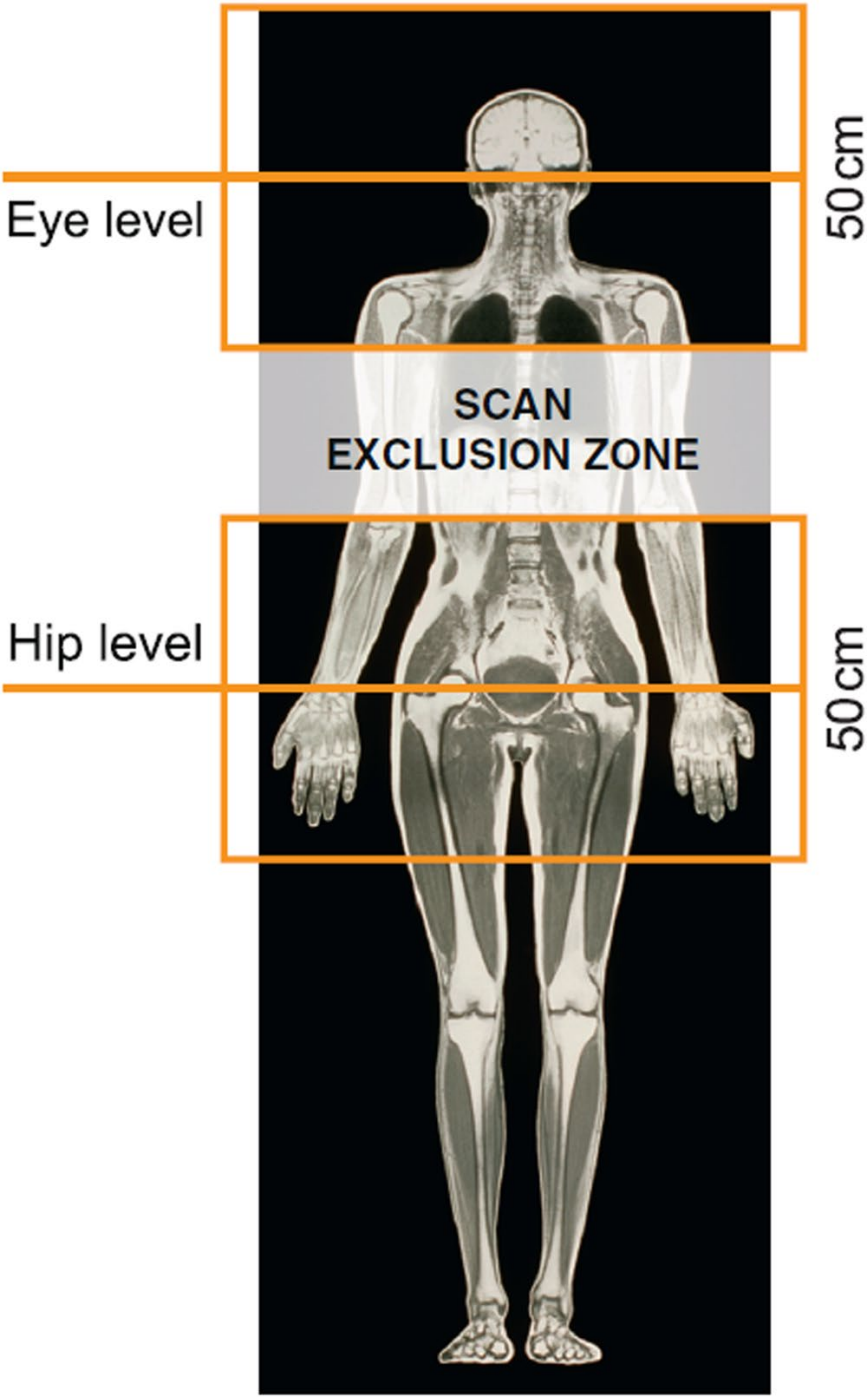
Isocentres at eye and hip level of magnetic resonance imaging scans, the available area for the scans (≈50 cm × 50 cm), and the scan exclusion zone. The size of the usually quadratic field of view varied with body region, scanner manufacturer (Siemens, Philips, GE), and the type of scan sequence (Table 2). The most common field of view was 23 cm × 23 cm, resulting in greater magnetic field gradients and thus more compelling testing conditions than it would be for a larger (e.g. 50 cm × 50 cm) field of view.

The patients were centred in the isocentre of the MRI system either at the level of the eyes (head scan) or at the trochanter (lower lumbar scan) (Fig. 2). The maximum slew rate and maximum specific absorption rate (SAR) were pre-specified in the study protocol (Table 2 footnote). All MRI sequences were designed to maximise SAR within the constraints of the “normal scanning mode”, i.e., maximum 2 W/kg for body MRI and maximum 3.2 W/kg for head scans. All head and lumbar sequences and all listed MRI scan conditions were tested and validated before study commencement on 1.5-T MRI systems from all three vendors (Siemens, Philips, GE) to ensure practicality.

After the MRI scans, the pulse generators were interrogated and the diagnostic data reviewed. The patients were assessed for adverse events, and lead evaluation was performed. All MRI images were reviewed for any relevant abnormalities. Device programming was restored to initial parameters or modified at the discretion of the investigator, including reactivation of ICD therapies. Enabling of ventricular capture control was recommended to allow transmission of threshold values via Home Monitoring.

### Study endpoints

Three primary endpoints were used to evaluate the safety and performance of the implanted systems under the MRI scanning conditions of the study protocol.

Primary endpoint 1 was defined as freedom from serious adverse device effects (SADEs) between the pre-scan MRI-mode programming and the 1-month post-MRI visit, related or possibly related to both the MRI procedure and the implanted system. An SADE-free rate >90% was considered success, calculated as 100% × (1 − number of SADEs divided by the number of MRI procedures). The SADE-free rates were determined separately for eight pulse generator categories: single-chamber ICD with DF-1 connector, single-chamber ICD with DF-4 connector, dual-chamber ICD/DF-1, dual-chamber ICD/DF-4, ICD DX/DF-1, CRT-D/DF-1, CRT-D/DF-4, and CRT-P, and for four lead categories: ICD lead DF-1, ICD lead DF-4, Solia, and Corox, defined in Table 1.

An independent external Data Safety Monitoring Board, composed of three physicians named in the Supplementary Information File, adjudicated whether adverse device effects (ADEs) and SADEs were related to MRI. SADEs were defined as ADEs that are categorised as serious adverse events, i.e. adverse events related to the ICD/CRT-P system and leading to death, hospitalisation, life-threatening condition, or potentially requiring implanted system revision or replacement.

Primary endpoints 2 and 3 evaluated pacing threshold increase and sensing amplitude decrease at one month post-MRI compared to the pre-MRI value, based on Home Monitoring data (if not available, then on in-office data). The four lead categories were evaluated separately to reflect RV values (ICD lead DF-1, ICD lead DF-4, Solia in the RV), left ventricular (LV) values (Corox), and RA values (Solia in the RA). A difference of log-transformed data for one month post-MRI vs. pre-MRI of ≤0.10 (pacing threshold) or ≥−0.10 (sensing amplitude) was regarded as non-appreciable alteration. The log-transformation was used to ensure statistical testing on normally distributed data.

Additional data of interest were pacing impedance, painless shock impedance, atrial sensing in ICD DX, battery status, and the success rate of ICD therapies.

### Study hypothesis and sample size calculation

The primary hypothesis H1 comprised all three primary endpoints pertaining to the ICD/CRT-D systems with DF-1 connector or to the CRT-P systems. The primary hypothesis H2 comprised all primary endpoints pertaining to the ICD/CRT-D systems with DF-4 connector. For either H1 or H2, the null hypotheses (“not MRI safe”) would be rejected if the respective SADE-free rates were >90% individually for each device category involved, and if the difference in log-transformed data were ≤0.10 for pacing threshold and ≥−0.10 for sensing amplitude for each lead category involved.

The calculation of the sample size was based on all primary endpoints and was a result of two alternative optimisation strategies, an interactive power calculation program and a gradient search algorithm. The optimised sample size combinations without drop-outs required 62 patients for each of the eight pulse generator categories, except for CRT-Ds with DF-1 (79 patients) or DF-4 (79 patients). The total required sample size was therefore 530 patients (=6 × 62 pts + 2 × 79 pts), or 590 patients including a 10% projected drop-out rate.

### Statistical analysis

Data were evaluated according to per-protocol analysis. The SADE-related sub-hypotheses were tested by exact binomial tests. Other sub-hypotheses were analysed by one-sample t-tests of the logarithm (base 2) of the data. Continuous variables are shown as means with standard deviation, median, and minimum/maximum. Categorical data are presented as absolute and relative frequencies. The 95% confidence intervals (CIs) are given for all primary endpoints. The analyses were carried out with SAS 9.4 (SAS Institute Inc., Cary, NC, USA) statistical software.

## Results

One hundred ninety-four patients were enrolled from June 20, 2013, to September 26, 2014 at 22 sites in Australia, Canada, Germany, Austria, France, Switzerland, Czech Republic, and Hungary (Supplementary Information File). The sponsor decided to stop the enrolment prematurely (see Supplementary Information File). The decision to stop the enrolment was not in any way related to safety issues. The premature termination resulted in an underpowering of the statistical analyses. Nevertheless, precisely defined MRI scans of this large patient cohort at the maximum magnetic resonance exposure burden provide strong corroboration related to safety aspects and electrical behavior of 146 ICD and pacemaker devices.

### Analysis population

Three quarters of the enrolled patients (n = 146) underwent the study-specific MRI and constituted the analysis population described in Table 3. The reasons for not undertaking MRI in 48 patients were pre-procedure exclusion criteria (n = 29), consent withdrawal (n = 14, in 3 cases due to patient anxiety or claustrophobia before or during MRI scans), the investigator’s decision to renounce MRI (n = 3), and loss to follow-up (n = 2).

**Table 3:** Patient characteristics (analysis population^a^).

Demographics	Results (n = 146)
Age at enrolment, years Mean ± SD Minimum, median, maximum	58.6 ± 14.3 20, 60, 85
**Gender**	
Male	117 (80.1%)
Female	29 (19.9%)
Pacing dependency	2 (1.4%)
Indication for ICD	142 (97.3%)
Primary prevention indication	96 (65.8%)
Secondary prevention indication	46 (31.5%)
Planned for CRT	30 (20.5%)
**Cardiovascular history**	
Coronary artery disease	73 (50.0%)
Cardiomyopathy	119 (81.5%)
Hypertension	83 (56.8%)
Valvular disease	15 (10.3%)
**Comorbidities**	
Diabetes mellitus	29 (19.9%)
Renal insufficiency	14 (9.6%)
Chronic pulmonary disease	13 (8.9%)
**Drugs affecting pacing threshold**	
Class I antiarrhythmics	1 (0.7%)
Class II antiarrhythmics	120 (82.2%)
Class III antiarrhythmics	18 (2.3%)
Class IV antiarrhythmics	2 (1.4%)

^a^Of 194 patients enrolled, 146 underwent the study-specific MRI (analysis population) and 48 did not undergo MRI because of pre-procedure exclusion criteria (n = 29), consent withdrawal (n = 14), or other reasons (n = 5, see text).

CRT: cardiac resynchronisation therapy; ICD: implantable cardioverter-defibrillator; MRI: magnetic resonance imaging; SD: standard deviation.

Table 4 shows the number of implanted devices from each category contributing separately to the primary hypotheses. The most common system was a dual-chamber ICD (n = 51), followed by a single-chamber ICD (n = 41), CRT-D (n = 27), ICD DX (n = 23), and CRT-P (n = 4). Nearly all pulse generators were placed in the left pectoral region (98%). The lead categories “ICD lead DF-1” and “ICD lead DF-4” were used in 76 and 66 patients, respectively. The Solia S lead was also well-represented (85 patients; 81 leads positioned in RA, 4 in RV). RA leads were mostly positioned in the RA appendage (85%), RV leads in the RV apex (52%) or at septum (42%), and LV leads (Corox) postero-laterally (50%) or laterally (47%).

**Table 4:** MRI-related SADE-free rate for predefined device categories (primary endpoint 1).

Device category (analysis population)	No. of devices	No. of SADEs	SADE-free rate	95%-CI^a^	Contributing to hypothesis^b^
**Pulse generator**					
Single-chamber ICD (DF-1)	18	0	100%	81.5–100%	H1
Single-chamber ICD (DF-4)	23	0	100%	85.2–100%	H2
Dual-chamber ICD (DF-1)	25	0	100%	86.3–100%	H1
Dual-chamber ICD (DF-4)	26	0	100%	86.8–100%	H2
ICD DX	23	0	100%	85.2–100%	H1
CRT-D (DF-1)	10	0	100%	69.2–100%	H1
CRT-D (DF-4)	17	0	100%	80.5–100%	H2
CRT-P	4	0	100%	39.8–100%	H1
**Lead**					
ICD lead DF-1^c^	76^c^	0^c^	100%^c^	95.3–100%^c^	H1
ICD lead DF-4^d^	66^d^	0^d^	100%^d^	94.6–100%^d^	H2
Solia	85	0	100%	94.8–100%	H1, H2
Corox	30	0	100%	88.4–100%	H1, H2

^a^The lower limit of the 95%-CI had to be >90% to reject the null hypothesis for a device category.

^b^H1 and H2 are predefined composite primary hypotheses (see Methods). In addition to SADE-free rate, also primary endpoints 2 and 3 (Table 5) contribute to H1 and H2.

^c,d^The results are also valid for the entire ICD/CRT-D system with DF-1 (^c^) or DF-4 (^d^) connection (post-hoc analysis).

CI: confidence interval; CRT-D/-P: cardiac resynchronisation therapy defibrillator/pacemaker; DF-1: old-standard connector type; DF-4: new-standard connector type; DX: ICD lead allowing atrial sensing via floating dipole; ICD: implantable cardioverter-defibrillator; SADE: serious adverse device effect.

### MRI scan

Patients were included in the analysis irrespective of the adherence to the MRI procedure window (Fig. 1), provided that the minimum delay of 9 weeks post-implantation was respected. The mean time from implantation to MRI scan was 107 ± 48 days (median 92, maximum 330).

The programmed MRI pacing mode was V00 in 19 patients (13%), D00 in 13 (9%), and OFF in 114 (78%). The time between MRI mode programming and reactivation of the initial programming was 68 ± 26 minutes (median 61.5, range 40–202). Patient monitoring method was pulse oximetry in 131 patients (90%), ECG in 91 (62%), and blood pressure in 60 (41%); an average of 1.9 monitoring methods was used per patient. Siemens MRI systems were used in 87 patients (60%), Philips in 40 (27%), and GE in 19 (13%).

Minimum, average, and maximum mean (±SD) achieved SAR (W/kg) of head scan sequences was 0.1 ± 0.1 (AX diffusion), 0.8 ± 0.8 (all sequences), and 1.5 ± 0.5 (COR FSE T2), as well as 0.4 ± 0.2 (Reference scan), 1.1 ± 0.6 (all sequences), and 1.5 ± 0.5 (SAG T2) for lumbar scan sequences.

### Follow-up compliance

All patients completed the study protocol at the 6-month post-MRI visit, except for three who were unable to appear for this follow-up and one who withdrew informed consent. No patient died during the study. The time from MRI scan to study termination was 183 ± 20 days (median 183, range 130–294).

### Primary endpoints

No MRI-related SADE occurred between MRI programming and the 1-month post-MRI visit, resulting in a 100% (95%-CI: 97.5–100%) MRI-related SADE-free rate of all 146 ICD/CRT-D/CRT-P systems. However, due to the premature enrolment stop, the lower limit of the 95%-CI did not reach 90% in nine of the 12 predefined device categories (Table 4), disallowing the rejection of H1 and H2 null hypotheses.

Table 5 summarises the results for pacing threshold increase and sensing amplitude decrease from immediately before MRI to the 1-month post-MRI visit, including primary endpoints 2 and 3. The Corox lead failed to pass the statistical test for both of these endpoints because of an underpowered group size (n = 28) compared to the other three lead categories (n = 62–79). The Solia lead narrowly missed the predefined threshold of ≥−0.10 for the sensing amplitude. For the primary endpoints 2 and 3, the four Solia leads implanted in the RV were moved from Solia to the “ICD lead DF-1” category. In this way, RV measurements with Solia were pooled with RV measurements with ICD leads for RV pacing thresholds, sensing amplitudes, and pacing impedances, whereas the results for the Solia lead category consists of RA values only.

**Table 5:** Pacing threshold and sensing amplitude changes at 1 month vs. pre-MRI (primary endpoints 2&3).

Lead category	No. of leads^a^	Mean ± SD	Median	Minimum, maximum	95%-CI (component of hypothesis^b^)
PT change, V @ 0.4 ms					
ICD lead with DF-1 (RV)^c^	78	0.0 ± 0.1	0.0	−0.2, +0.2	—
ICD lead with DF-4 (RV)	65	0.0 ± 0.1	0.0	−0.5, +0.2	—
Solia (RA)^d^	79	0.0 ± 0.1	0.0	−0.3, +0.5	—
Corox (LV)	28	0.0 ± 0.2	0.0	−0.5, +0.5	—
Primary endpoint 2 (change in log-transformed PT)					Upper limit:^e^
ICD lead with DF-1 (RV)^c^	78	0.0 ± 0.2	—	−0.3, +0.5	0.06 (H1)
ICD lead with DF-4 (RV)	65	0.0 ± 0.2	—	−0.7, +0.6	0.07 (H2)
Solia (RA)^d^	79	0.0 ± 0.3	—	−0.6, +1.2	0.08 (H1, H2)
Corox (LV)	28	0.0 ± 0.2	—	−0.5, +0.7	0.14 (H1, H2)
SA change, mV					
ICD lead with DF-1 (RV)^c^	74	−0.1 ± 1.7	0.0	−5.8, +8.9	—
ICD lead with DF-4 (RV)	62	0.0 ± 1.1	0.0	−4.1, +3.0	—
Solia (RA)^d^	67	0.0 ± 0.6	0.0	−3.3, +2.0	—
Corox (LV)	28	−0.2 ± 3.0	0.2	−12.7, +3.7	—
Primary endpoint 3 (change in log-transformed SA)					Lower limit:^f^
ICD lead with DF-1 (RV)^c^	74	0.0 ± 0.2	—	−0.4, +1.1	−0.05 (H1)
ICD lead with DF-4 (RV)	62	0.0 ± 0.1	—	−0.4, +0.4	−0.02 (H2)
Solia (RA)^d^	67	0.0 ± 0.3	—	−2.1, +0.8	−0.11 (H1, H2)
Corox (LV)	28	0.0 ± 0.4	—	−1.9, +0.4	−0.20 (H1, H2)

^a^Leads with both 1-month and pre-MRI result available. Only measurement pairs in the same polarity and with the same pulse width are taken into account.

^b^H1 and H2 are predefined composite primary hypotheses (see Methods). In addition to SADE-free rate (Table 4), also primary endpoints 2 and 3 contribute to H1 and H2.

^c^Including 4 Solia leads positioned in the right ventricle.

^d^Excluding 4 Solia leads positioned in the right ventricle.

^e^Upper limit ≤ 0.10 was needed to reject the null hypothesis.

^f^Lower limit ≥ −0.10 was needed to reject the null hypothesis.

CI: confidence interval; DF-1: old-standard connector type; DF-4: new-standard connector type; ICD: implantable cardioverter-defibrillator; LV: values for left ventricle; PT: pacing threshold; RA: values for right atrium; RV: values for right ventricle; SA: sensing amplitude; SD: standard deviation.

### Events related to MRI

While there were no MRI-related SADEs or complications, three events were classified as possibly MRI-procedure related: (1) feeling of implant site warmth and mild pain in the left shoulder (dual-chamber ICD/DF-1), (2) feeling of implant site warmth and impression that one electrode was warmer (CRT-P), and (3) a low-intensity vibration around the ICD pocket and implant site paresthesia (dual-chamber ICD/DF-1). These temporary sensations did not require any action, and the MRI scanning continued. Subsequent device checks in these three individuals were unremarkable.

### Other findings

The pacing impedance at pre-MRI vs. one month post-MRI was 528 ± 82 vs. 525 ± 86 Ω (RV), 719 ± 183 vs. 762 ± 170 Ω (LV), and 569 ± 72 vs. 578 ± 68 Ω (RA). Painless shock impedance was 70 ± 11 vs. 71 ± 12 Ω. P-wave amplitude in the ICD DX systems was 6.5 ± 5.7 vs. 6.3 ± 5.6 mV. Battery status in all 146 devices was 100% at one month post-MRI.

The success rate of ICD therapies was calculated for ventricular tachyarrhythmia occurring within 6 months after MRI, excluding episodes that were induced, that were detected in the monitoring zone not requiring treatment, that ended spontaneously, or that were terminated externally before the device could deliver any shocks. A total of 33 episodes met the inclusion criteria, 32 of which were terminated successfully by the ICD. One episode of monomorphic ventricular tachycardia with short ventriculo-atrial interval was misclassified as supraventricular, and ICD therapy was withheld inappropriately.

Although the study-specific MRI scans were conducted without clinical indication, all MRI images were reviewed for any incidental findings. In 17 patients, abnormalities were noted (see Supplementary Information File). Systematic evaluation of MRI image quality or distortions was not performed within the present study.

## Discussion

Although the premature enrolment stop did not allow statistically significant findings, there was no evidence of harm to the patients or incorrect function of the investigated MRI-conditional systems during head and lower lumbar MRI with 1.5-T scanners. These findings are consistent with previously published reports on MRI in patients with pacemaker/ICD systems specifically designed or modified for the MRI environment.

Despite increasing numbers of patients with MRI-conditional devices and very low reported complication rates, provision of MRI in these patients is currently poor as has been investigated by Sabzevari *et al*.^14^. The concerns about safety of MRI in device patients, a lack of training, and logistical difficulties were stated as the main reasons for not performing MRI scans on patients with MRI-conditional devices^14^. Also Celentano *et al*. reported low MRI examination rates and frequent denial of MRI exams despite MRI-conditional implants^15^. Our study considerably adds to the increasing evidence on safety: 146 patients with implanted MRI-conditional pacemakers and ICD systems have been examined, with no evidence of harm to the patients or any negative influence of the MRI procedure on the implanted system.

To our knowledge, clinical studies on MRI-conditional devices published in the scientific literature up to March, 2019 include eight pacemaker studies^3–7,9,18,19^, three ICD studies^8,10,11^, and two studies with both pacemakers and ICDs^15,20^. These 13 previous studies involved altogether 1258 pacemaker and 343 ICD patients who underwent MRI and were followed for ≥1 month. Eleven of the thirteen studies reported absence of any MRI-related SADE. In one study, a pacemaker patient had pericarditis with pericardial effusion without evidence of perforation, which was classified as an SADE possibly related to a thoracic MRI^9^. In another study, two complications included (i) diaphragmatic stimulation when the device was switched to MRI-conditional mode resulting in scan abandonment and (ii) device failure post-MRI requiring manufacturer reprogramming^20^.

Minor sensations during MRI, such as implant site warmth, paresthesia, or vibration, reported by three patients (2.1%) in our study required no action and appeared to be a minor issue compared to the potential benefit of MRI in device patients. By comparison, Gimbel *et al*.^5^ reported five events of this kind in 150 pacemaker patients undergoing MRI (3.3%), Wilkoff *et al*.^3^ mentioned eight events in 211 pacemaker patients (3.8%), and Gold *et al*.^11^ observed five events in 156 ICD patients (3.2%). The other studies did not report minor sensations systematically.

ProMRI PROVEN was the first study to include MRI-conditional CRT-D and CRT-P devices and, thus, coronary sinus leads (n = 30) that have looser fixation than ICD, ventricular, and atrial leads with a screw-in mechanism, and may therefore be more prone to mechanical movement in electromagnetic fields. However, no problems were noticed.

Overall, ProMRI PROVEN is the fourth multicentre study of Biotronik MRI-conditional devices subjected to 1.5-T MRI tests. The previous three, mainly US-based studies showed MRI safety in ICD patients for thoracic spine and cardiac scans (n = 148; Iforia ICD platform; Awad *et al*.^8^), in pacemaker patients for head and lower lumbar scans with exclusion zone (n = 229; Evia/Entovis pacemaker platforms; Bailey *et al*.^7^), and in pacemaker patients for thoracic spine and cardiac scans (n = 221; Entovis platform; Bailey *et al*.^9^). The rest of Biotronik MRI-conditional studies were single-centre, including 30 or less patients^4,10^. In contrast to all these previous studies, ProMRI PROVEN evaluated the greatest number of different pulse generator and lead models, and MRI tests were performed in most diverse geographies and clinical environments with highly-variable scanning experience (22 sites in Australia, Canada, and six European countries). We deem that this versatility of materials and methods theoretically increased the chance for adverse events to occur compared with more uniform conditions for head and lower lumbar scans. Still, no SADE was observed, despite higher maximum SAR exposure allowed during head scans (3.2 W/kg) and longer total scan time (≈30 minutes) than in the past (due to more sequences tested).

Unlike several other studies, ProMRI PROVEN imposed an exclusion zone on the thoracic region. However, the majority of clinically indicated MRI scans are focused on either the head or the abdominal regions.

Apart from MRI-conditional devices, several studies evaluated the impact of 1.5-T MRI on non-conditional cardiac devices. One study specifically examined patients with established contraindications for MRI scans such as abandoned leads, to find significant changes in battery voltage and lead impedance immediately after the MRI scans^21^. A recently published multicentre MagnaSafe Registry analysed the results of 818 pacemaker and 428 ICD patients undergoing clinically indicated nonthoracic MRI scans despite having conventional non-MRI-conditional devices^22^. Among patients scanned according to the study set up, the most common effect was a minor change in ICD shock impedance and changes in the device setting without clinical significance^22^. Another multicentre registry analysed the results of 880 pacemaker and 629 ICD patients undergoing clinically indicated thoracic and nonthoracic MRI scans with non-MRI-conditional devices, to observe rare cases of device reset to a back-up mode and changes in lead parameters without clinical significance^23^. Albeit these encouraging results have influence on national guidelines, they do not equal a technical proof of device safety^24,25^. Despite the relatively low risk of adverse events for non-conditional devices, a potential risk remains even under defined conditions.

### Study limitations

Due to the premature enrolment stop, only 33% of the projected study cohort was enrolled (194/590), resulting in study underpowering and non-rejection of the null hypotheses. Irrespective of the number of enrolled patients, clinical studies generally do not have the power to detect rare safety events, and they do not supplant the need for postmarket surveillance.

The use of MRI scanners was limited to well-defined conditions and safe use in other conditions has not been demonstrated. The exclusion zone used in this study was a common approach at the time of study design. MRI artefacts associated with the implanted system^1,26,27^ were not analysed. Evaluation of MRI image quality and distortions is indispensable for thoracic and cardiac MRI scans because pulse generators in the field of view can cause significant artefacts hampering diagnostic value of the images, especially for tissues immediately adjacent to the pulse generator^1,8,9,14,18,26–29^. In our study, pulse generators and leads were outside the fields of view for both head and lower lumbar scans. Since no clinically important artefacts under these conditions were observed in previous studies^1,4,14,27^, we did not evaluate image quality systematically. All MRI images were, however, reviewed for any incidental medical findings and no concerns regarding degradation of image quality were raised.

### Perspectives

In the meantime, MRI-conditional systems are available that are suitable for full-body scans at 1.5 T and 3 T. Apart from technological progress, increasing attention is being paid to the clinical workflow. At present, prior to the MRI scan a cardiologist must program the device to an MRI-conditional mode with reduced functionality (e.g., deactivated tachyarrhythmia therapies). After the scan, the cardiologist again programs the device to restore full functionality. This workflow requires availability of both a cardiologist and radiologist to reduce the patient’s time in the MRI mode. Recently developed automatic MRI functions (Biotronik, Sorin/Livanova) enable the devices to automatically detect an MRI field, switch to the pre-specified MRI mode, and restore original functionality after detecting MRI termination. In preparation for the MRI, the cardiologist must activate this function for a certain time window. Such procedural improvements minimise the time in which the device functionality is limited, simplify the clinical workflow, and lower the infrastructural requirements for conducting MRI scans in pacemaker and ICD patients.

## Conclusions

Although the premature enrolment stop did not allow statistically significant findings, there was no indicator of incorrect function of the investigated Biotronik ProMRI ICD and CRT-D/-P systems in patients undergoing 1.5-T head and lower lumbar MRI under consideration of the exclusion zones and other MRI conditions as used in this trial. The data were collected in diverse clinical environments (sites from Australia, Canada and six European countries). The results of this trial should promote further acceptance of performing clinically indicated MRI procedures in patients with MRI-conditional devices.

## Supplementary information

Supplementary information

## Data Availability

The data that support the findings of this study are available from Biotronik but restrictions apply to the availability of these data, which were used under license for the current study, and are therefore not publicly available. Data are, however, available from the authors upon reasonable request and with permission of Biotronik.
