# Supplementary material for: Clinical safety of ProMRI implantable cardioverter-defibrillator systems during head and lower lumbar magnetic resonance imaging at 1.5 Tesla
Source: Sci Rep. 2019 Dec 3;9:18243. doi: 10.1038/s41598-019-54342-4 (PMC6890633; doi:10.1038/s41598-019-54342-4)
Supplement: Supplementary file 1 — Supplementary information [file 41598_2019_54342_MOESM1_ESM.pdf]

Supplementary information

Article title:

Clinical safety of ProMRI implantable cardioverter-defibrillator systems during head and lower lumbar magnetic resonance imaging at 1.5 Tesla

Authors:

Wolfgang Bauer, Dennis H. Lau, Christian Wollmann, Andrew McGavigan, Jacques Mansourati, Theresa Reiter, Simone Frömer, Mark E. Ladd, Harald H. Quick

Table of contents

Principal investigators and investigational sites ………………………………….... 2

Clinical Coordinating Investigator, Scan Advisory Committee, Data Safety Monitoring Board …………………………………………………………………………...... 3

Patient inclusion and exclusion criteria ……………………………………………… 4

Premature study termination …………………………………………………………... 5

Incidental findings in magnetic resonance images……………………………….. 6

Principal investigators and investigational sites

(alphabetical order of countries and investigators)

Australia (6): Gemma Figtree (**city:** St Leonards, **institution:** Royal North Shore Hospital); Angas Hamer (Box Hill, Box Hill Hospital); Andrew McGavigan (Bedford Park, Flinders Medical Centre); Vince Paul (Perth, Royal Perth Hospital); Prashanthan Sanders (Adelaide, Royal Adelaide Hospital); Raymond Sy (Camperdown, Royal Prince Alfred Hospital);

Austria (2): Clemens Steinwender (Linz, Allgemeines Krankenhaus der Stadt Linz GmbH); Christian Wollmann (St. Pölten, St. Pölten Landesklinikum);

Canada (5): Felix Ayala-Paredes (Sherbrooke - Quebec, Centre Hospitalier Universitaire de Sherbrooke); Douglas Cameron (Toronto - Ontario, Toronto General Hospital); Eugene Crystal (Toronto - Ontario, Sunnybrook Health Sciences Centre); Katia Marjolaine Dyrda (Montreal - Quebec, Montreal Heart Institute); Randall Williams (Edmonton - Alberta, Royal Alexandra Hospital);

Czech Republic (1): Miloš Táborský (Olomouc, University Hospital Olomouc);

France (2): Dominique Babuty (Tours cedex 9, CHU Tours - Hôpital Trousseau); Jacques Mansourati (Brest Cedex, CHU Brest - Hôpital de la Cavale Blanche);

Germany (4): Wolfgang Bauer (Würzburg, Universitätsklinikum Würzburg); Johannes Brachmann (Coburg, Klinikum Coburg); Werner Jung (Villingen-Schwenningen, Schwarzwald-Baar Klinikum); Jörg Otto Schwab (Bonn, Universitätsklinikum Bonn);

Hungary (1): Béla Merkely (Budapest, Semmelweis Medical University);

Switzerland (1): Rainer Zbinden (Zürich, Stadtspital Triemli).

Clinical Coordinating Investigator: Wolfgang Bauer (Universitätsklinikum Würzburg, Würzburg, Germany).

Scan Advisory Committee: Siemens scan parameters: Zbynék Tüdös (University Hospital, Olomouc, Czech Republic); Philips scan parameters: Atila Tóth (Semmelweis University Heart Center, Budapest, Hungary); GE scan parameters: Christopher Beynon (Institut für Radiologie, Zürich, Switzerland)

Data Safety Monitoring Board: Charles Henrikson (Oregon Health & Sciences University, Portland, OR, USA); Sei Iwai (Westchester Medical Center, Woods Rd, Valhalla, NY, USA); and Jeffrey Winterfield (Loyola University Medical Center, Maywood, IL, USA). The board also had the function of an Event Adjudication Committee regarding MRI-relatedness of adverse device effects reported by study investigators. The board members were not involved in the conduction of the study and were blinded to investigational sites.

Patient inclusion and exclusion criteria

Consenting patients were enrolled if they were >18 years old, had a standard indication for an ICD or a CRT-D/-P to be implanted in pectoral position, were ≥140 cm tall, were able and willing to attend all follow-up examinations and complete all testing required by the clinical protocol including one non-diagnostic MRI scan, and were able and willing to activate the Cardio Messenger for remote monitoring by Biotronik® Home Monitoring technology.[1-2]

Patients were not enrolled in case of short life expectancy (<8 months), planned cardiac surgery within 8 months from enrolment, pregnancy, persistent (>7 days) or permanent atrial tachyarrhythmia in conjunction with the intention to implant a device requiring an atrial lead, presence of metallic objects in the patient's body susceptible to interaction with MRI, or cardiovascular implants that contradict the conditions of the current Biotronik ProMRI® manual.

Enrolled patients were excluded from the MRI scan if any of the following prerequisites for MRI scan admission were not met: patient afebrile; implanted for at least 9 weeks; right atrial (RA), right ventricular (RV), and left ventricular (LV) pre-MRI pacing threshold ≤2.0 V @0.4 ms; lead impedance 200–1500 Ω; shock impedance 30–90 Ω; absolute value of the pacing threshold difference between the 8-week follow-up and the pre-MRI procedure ≤0.5 V for RA/RV leads and ≤1.0 V for the LV lead; no persistent or permanent atrial arrhythmia in a patient with an RA lead; and no cardiovascular implant not belonging to the pre-specified investigational devices.

References

1. Burri, H. & Senouf, D. Remote monitoring and follow-up of pacemakers and implantable cardioverter defibrillators. *Europace*. **11**, 701-709 (2009).
2. Hindricks, G. *et al*. Implant-based multiparameter telemonitoring of patients with heart failure (IN-TIME): a randomised controlled trial. *Lancet*. **384**, 583-590 (2014).

Premature study termination

The study was initially planned to enrol 590 patients, but the enrolment was prematurely stopped by the sponsor on September 19, 2014. The decision for the enrolment stop was not in any way related to safety issues of the devices.

There was cumulating evidence for the safety of MRI-conditional devices from literature, technical simulations, post-market surveillance reports, and other clinical studies leading to an increasing acceptance by regulatory authorities. Together with the already existing data from this trial the body of evidence for safety was large enough for regulatory approval, so that continuation of the study was not necessary.

Despite early termination, study results are being published in light of the current evidence indicating that MRI-conditional labelling of cardiac devices is still not accepted in segments of the clinical community because of residual safety concerns and a perceived lack of an adequate evidence base for safety.

Observed abnormalities in MRI images

Category of finding	Number of findings
Cyst	4
Renal cyst	2
Cerebral cyst	1
Old stroke (not always known)	7
Lumbar spine degeneration	3
Abnormality within the maxillary sinus	1
Vertebral marrow lesion	1
Metal susceptibility artefact	1
Gliosis	1
